# Low expression of PINK1 and PARK2 predicts poor prognosis in patients with esophageal squamous cell carcinoma

**DOI:** 10.1186/s12957-023-03206-3

**Published:** 2023-10-13

**Authors:** Xiangyun Lu, Yongkun Yao, Yandi Ma, Xudong Zhang, Hao Peng, Yuhui Pei, Yulin Lu, Lianghai Wang

**Affiliations:** 1 Department of Pathology and Key Laboratory for Xinjiang Endemic and Ethnic Diseases, the First Affiliated Hospital/Shihezi University School of Medicine, Shihezi, Xinjiang China; 2 Department of Pathology, Nanyang Central Hospital, Nanyang, Henan China; 3 Department of Pathology, the First Clinical Medical College of Weifang Medical University, Weifang People’s Hospital, Weifang, Shangdong China

**Keywords:** Esophageal squamous cell carcinoma, PINK1, PARK2, Prognosis

## Abstract

**Background:**

The Parkinson’s disease (PD) gene family expression is strongly linked to tumor development and progression; *PINK1* and *PARK2* are essential members of the PD gene family. However, the relationship between PINK1 and PARK2 and esophageal squamous cell carcinoma (ESCC) remains unknown. This research aims to clarify the prognostic value of PINK1 and PARK2 in ESCC.

**Methods:**

PINK1 and PARK2 protein levels in 232 ESCC specimens, and 125 matched adjacent normal tissues were detected by immunohistochemistry. The relationship between PINK1 and PARK2 protein expression and clinicopathological features were analyzed. Kaplan–Meier survival analysis was performed to estimate the prognostic value of the PINK1 and PARK2 proteins in patients. Cox univariate and multivariate analyses were used to assess the risk factors affecting the OS for patients with ESCC.

**Results:**

PINK1 and PARK2 had low expression in ESCC. Patients with low PINK1 had worse differentiation and advanced T and TNM stages. Lower PARK2 expression was linked to lymph node metastases and an advanced TNM stage. Furthermore, reduced PINK1 and PARK2 levels were associated with a poor prognosis for ESCC. Cox univariate and multivariate analyses revealed that PINK1, PARK2, and tumor size were closely associated with the prognosis of patients with ESCC, and PARK2 was an independent risk factor for patients with ESCC. Finally, the PINK1 and PARK2 proteins were closely related and shared the same signal pathway.

**Conclusions:**

PINK1 and PARK2 could work as tumor suppressors in ESCC and are likely to become new treatment targets for ESCC.

**Supplementary Information:**

The online version contains supplementary material available at 10.1186/s12957-023-03206-3.

## Introduction

Esophageal carcinoma (EC) is one of the top 5 cancers in terms of incidence and mortality in China and the leading cause of cancer deaths globally [1]. Esophageal squamous cell carcinoma (ESCC) is the most common type of EC, affecting 90% of patients [2]. Despite advances in diagnostic tools and multimodal therapy, ESCC survival rates remain low [3, 4]. Although the mechanism is unknown, many genes may be implicated, consistent with multiple tumorigenesis and malignant behavior elements [5–7]. The Parkinson’s disease (PD) gene family appears to affect cancer cell proliferation and migration [8–11].

The PD gene family—*PARK1, PARK2, PARK5, PARK6,* and *PARK7*—is crucial to developing PD [12, 13]. PD gene expression is also closely linked to tumor development and progression [14, 15]. The *PINK1* gene, also known as *PARK6*, encodes the 581-amino-acid PINK1 protein [16]. The *PINK1* gene is extensively expressed in mammalian organs and cells [17] and plays a crucial role in injury, inflammation, and other processes [15, 18]. *PINK1* induces mitochondrial dysfunction, oxidative stress, and cell death and promotes tumor growth. MYC regulates metabolic reprogramming in colorectal cancer by decreasing *PINK1* expression [19]. The *PINK1* gene knockdown may lower breast cancer cell malignancy [20]. Furthermore, ESCC models with strong PINK1 protein expression may tolerate neoadjuvant chemotherapy [21]. However, few studies have shown how PINK1 affects ESCC progression.

*PARK2*, also known as Parkin, is a neuroprotective gene in the PD gene family [22]. *PARK2* regulates the generation cycle, mitochondrial dynamic balance, energy metabolism, and other cellular activities [23–25] and is linked to many diseases. These findings suggest that it is an extensive and critical gene. The *PARK2* gene has been identified as a new cancer-associated factor in liver cancer [26], glioblastoma [27], lung cancer [28], and colon cancer cell lines [29]. PARK2 overexpression can inhibit these cells from reproducing. PARK2 inhibition increased pancreatic cancer cell proliferation and tumorigenicity in vitro [30]. PARK2 expression and prognosis in ESCC remain unknown.

We investigated the expression of the PINK1 and PARK2 proteins in ESCC using immunohistochemistry. The clinicopathological characteristics of patients with ESCC were linked to the expression of PINK1 and PARK2. The prognostic value of PINK1 and PARK2 in patients with ESCC was investigated. Furthermore, the risk factors affecting the overall survival (OS) of patients with ESCC were analyzed using Cox univariate and multivariate analysis. The signal pathways *PINK1* and *PARK2* were also predicted to confirm their possible mechanism or pathway in ESCC development.

## Materials and methods

### Clinical specimens

A total of 232 radically resected ESCC tissues, 128 matched neighboring normal tissue specimens, and patients’ clinicopathological data were collected between 2013 and 2020, excluding those who had preoperative chemotherapy or radiotherapy. Patients were followed up via telephone or medical records until October 2, 2021, or death.

### Immunohistochemistry (IHC) and assessment

A tissue microarray was formed using 232 ESCC and 128 normal tissue specimen wax blocks. Immunohistochemical experiments were performed on 4-μm tissue microarray sections. Antigens were repaired using a tris–EDTA buffer (pH 9.0) with high-pressure heat recovery for 8 min. After dewaxing and repairing the sections, endogenous peroxidase was inhibited for 10 min by 3% H2O2 before being washed three times in 5 min with a phosphate-buffered saline (PBS) solution. The IHC was conducted on stained tissue sections using the EnVision method (DAKO, Glostrup, Denmark). The tissue sections were incubated for 8 h at 4 °C with anti-PINK1 rabbit polyclonal antibody (1:1000 dilution; 23,274–1-AP; Proteintech; China) and anti-PARK2 rabbit polyclonal antibody (1:800 dilution; 14060–1-AP; Proteintech; China). The sections were washed with PBS solution and incubated with the second EnVision antibody for 30 min at 37 °C. Finally, proteins were detected using 3,3′-diaminobenzidine and hematoxylin. The tissue slices were counterstained with hematoxylin and eosin and observed under optical microscopy.

The product of the percentage of positive cells and staining intensity yielded four grades of staining scores: “0” for negative, “1–4” for weak, “5–6” for moderate, and “7–12” for strong. The scores of positive cells were defined as follows: “0” denotes 0–5% positive cells; “1” denotes 6–25% positive cells; “2” denotes 26–50% positive cells; “3” denotes 51–75% positive cells; and “4” denotes ≧ 76% positive cells. The staining strength grades were 0 (no staining), 1 (yellow), 2 (brown-yellow), and 3 (brown). Two pathologists examined the staining intensities and positive cell proportions; if there was a disagreement between the two pathologists, a third pathologist was invited to investigate. The low-expression group scored ≦ 6, whereas the high-expression group scored > 6.

### Gene set enrichment analysis (GSEA)

GSEA was used to investigate the biological signaling pathway of *PINK1* and compare the low- and high-expression groups to the median expression level. As mentioned above, the *PARK2* signaling pathway was investigated. The net enrichment score (NES), gene ratio, and *P* value determined the enrichment items of the Kyoto Encyclopedia of Genes and Genomes (KEGG) pathways. We set the random sample permutation number to 178, and significant enrichment was characterized as gene sets with default values (|NES|> 1, NOM *P* < 0.05, and FDR *q* value < 0.25).

### Statistical analysis

All statistical analyses were conducted using SPSS 25.0. Pearson’s chi-square analysis was used to compare PINK1 and PARK2 expression in ESCC and normal tissues and to determine the relationship between PINK1 and PARK2 expression with clinicopathological features in ESCC. To estimate the prognostic value of the PINK1 and PARK2 proteins in patients, a Kaplan–Meier survival analysis was performed. The relationship between the PINK1 and PARK2 proteins was then analyzed using correlation analysis. Furthermore, Cox univariate and multivariate analyses were used to assess the risk factors affecting the OS for patients with ESCC. The significance of a difference was set at* P* < 0.05.

## Results

### PINK1 and PARK2 low expression was associated with tumor malignancy

**Fig. 1:**
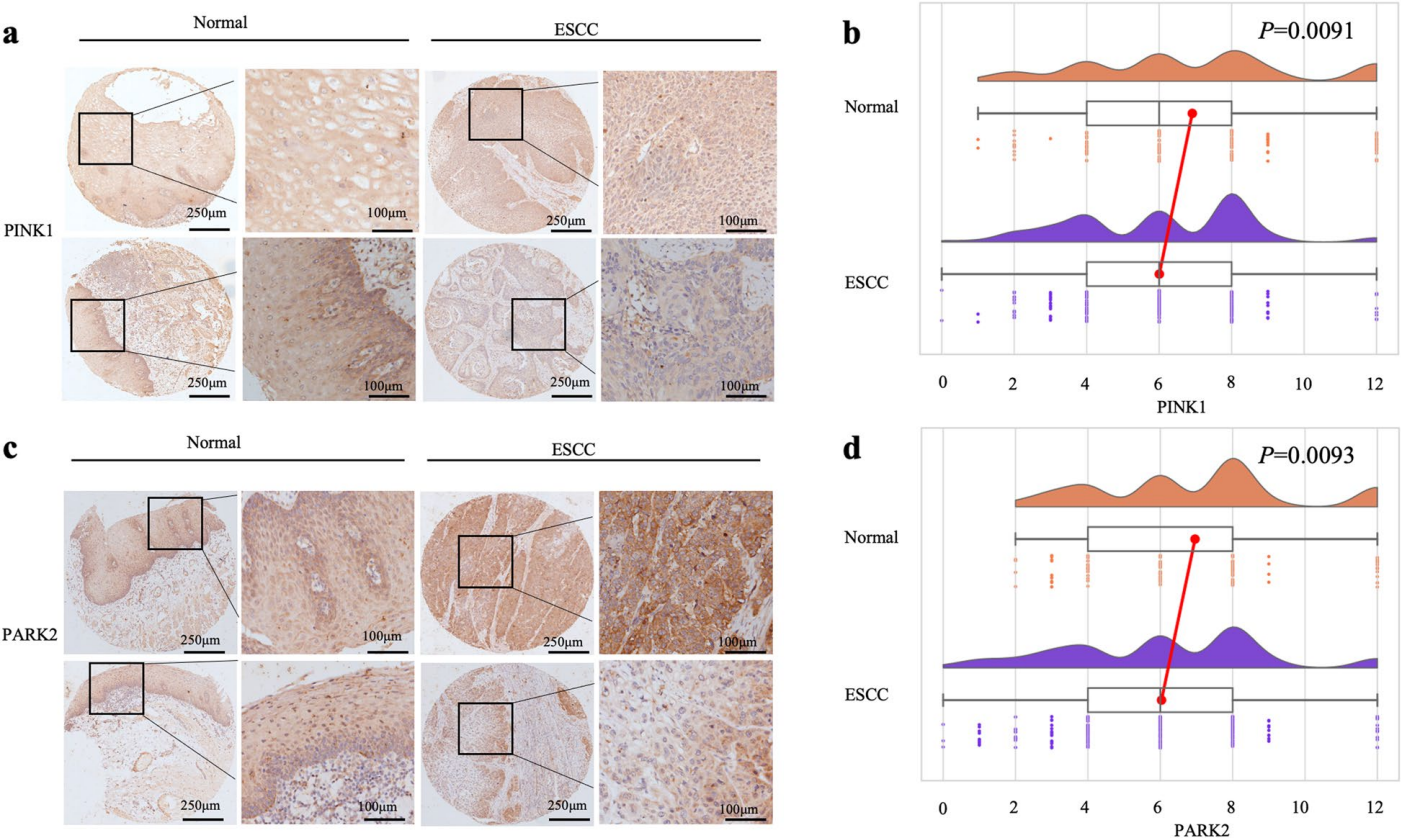
Low expression of PINK1 and PARK2 in ESCC tissues. **a** The PINK1 protein is present in the cytoplasm of normal and ESCC tissues, strongly expressed in normal tissues, and weakly expressed in ESCC tissues (magnification: × 40 or × 200). **b** The PINK1 protein immunohistochemical score distributions in normal and ESCC tissues are statistically different (*P* < 0.05). **c** The PARK2 protein is present in the cytoplasm of normal and ESCC tissues, where it is strongly expressed in normal tissues and weakly expressed in ESCC tissues (magnification: × 40 or × 200). **d** The PARK2 protein immunohistochemical score distributions in normal and ESCC tissues are statistically different (*P* < 0.05)

We immunostained 128 normal and 232 ESCC tissue samples. The PINK1 and PARK2 proteins were primarily found in the cytoplasm of normal tissues and ESCC samples (Fig. 1a and c). The ratio of PINK1 low expression in ESCC tissues (62.5%, 145/232) was significantly higher than that in normal tissues (51.6%, 66/128) (*P* < 0.05; Table 1 and Fig. 1b). Similarly, the ratio of PARK2 low expression in ESCC tissues (60.8%, 141/232) was also higher than that in normal tissues (48.4%, 62/128) (*P* < 0.05; Table 1 and Fig. 1c and d). Therefore, ESCC had low PINK1 and PARK2 expressions.

**Table 1:** The expression of PINK1 and PARK in ESCC and adjacent normal tissues

Group	PINK1		PARK2	
	Normal *n* (%)	ESCC *n* (%)	Normal *n* (%)	ESCC *n* (%)
Low expression	66 (51.6)	145 (62.5)	62 (48.4)	141 (60.8)
High expression	62 (48.4)	87 (37.5)	66 (51.6)	91 (39.2)
χ2	4.068 0.044^a^		5.106 0.024^a^	
*P*				

^a^Statistically significant difference

The relationship between PINK1, PARK2, and clinicopathological manifestations in patients with ESCC was investigated using Pearson’s chi-square analysis. PINK1 expression in ESCC differed statistically by grade, T stage, and TNM stage. The ESCC with low PINK1 expression differentiated worse than the ESCC with high PINK1 expression (*P* = 0.029, Table 2). T3–T4 stage patients with ESCC had lower PINK1 levels than T1–T2 stage patients with ESCC (*P* = 0.018, Table 2), whereas advanced TNM stage patients with ESCC had lower PINK1 levels (*P* = 0.022). In patients with ESCC, PINK1 expression was not affected by age, sex, tumor size, location, lymph node metastasis, or metastasis (*P* > 0.05, Table 2). In ESCC, PARK2 protein expression was associated with lymph node metastases and TNM stage (*P* < 0.05, Table 2). Patients with lymph node metastases had lower PARK2 levels (*P* = 0.039, Table 2). Patients with an advanced TNM stage had lower PARK2 expression (*P* = 0.040, Table 2). The sex, age, tumor size, location, and metastasis of patients with ESCC were unrelated (*P* > 0.05, Table 2).

**Table 2:** Relationship between the expression of PINK1 and PARK2 and clinicopathological characteristics of the ESCC patients

Parameters	*n*	PINK1				PARK2			
		Low *n* (%)	High *n* (%)	χ^2^*/t*	*P value*	Low *n* (%)	High *n* (%)	χ^2^/*t*	*P value*
Sex				0.296	0.587			1.080	0.303
Male	157	100 (63.7)	57 (36.3)			99 (63.1)	58 (36.9)		
Female	75	45 (60.0)	30 (40.0)			42 (56.0)	33 (44.0)		
Age				0.117	0.733			0.296	0.586
< 60	102	65 (63.7)	37 (36.3)			64 (62.7)	38 (37.3)		
≥ 60	130	80 (61.5)	50 (38.5)			77 (59.2)	53 (40.8)		
Location				0.420	0.517			3.190	0.074
Up and middle	129	83 (64.3)	46 (35.7)			85 (65.9)	44 (34.1)		
Low	103	62 (60.2)	41 (39.8)			56 (54.4)	47 (45.6)		
Tumor size				1.797	0.180			0.966	0.326
≤ 3 cm	68	38 (55.9)	30 (44.1)			38 (55.9)	30 (44.1)		
> 3 cm	164	107 (65.2)	57 (34.8)			103 (62.8)	61 (37.2)		
Grade^a^				7.107	0.029*			1.551	0.460
G1	35	15 (42.9)	20 (57.1)			18 (51.4)	17 (48.6)		
G2	148	96 (64.9)	52 (35.1)			93 (62.8)	55 (37.2)		
G3	49	34 (69.4)	15 (30.6)			30 (61.2)	19 (38.8)		
T stage				5.623	0.018*			1.823	0.177
T1–T2	97	52(53.6)	45(46.4)			54 (55.7)	43 (44.3)		
T3–T4	135	93(68.9)	42(31.1)			87 (64.4)	48 (35.6)		
Lymph node metastasis				3.231	0.072			4.265	0.039*
N0	113	64 (56.6)	49 (43.4)			60 (53.1)	52 (46.9)		
N1	119	81 (68.1)	38 (31.9)			80 (67.2)	39 (32.7)		
Metastasis				0.932	0.334			1.135	0.287
M0	223	138 (61.9)	85 (38.1)			134 (60.1)	89 (39.9)		
M1	9	7 (77.8)	2 (22.2)			7 (77.8)	2 (22.2)		
TNM stage^b^				5.225	0.022*			4.230	0.040*
I-II	103	56 (54.4)	47 (45.6)			55 (53.4)	48 (46.6)		
III-IV	129	89 (69.0)	40 (31.0)			86 (66.7)	43 (33.3)		

^a^Histologic grade was based on the WHO classification published in 2019

^b^TNM stage was assessed according to the 9th Edition of the AJCC Cancer Staging Manual

^*^Statistically significant difference

### Low expression of PINK1 and PARK2 predicted poor prognosis in ESCC

**Fig. 2:**
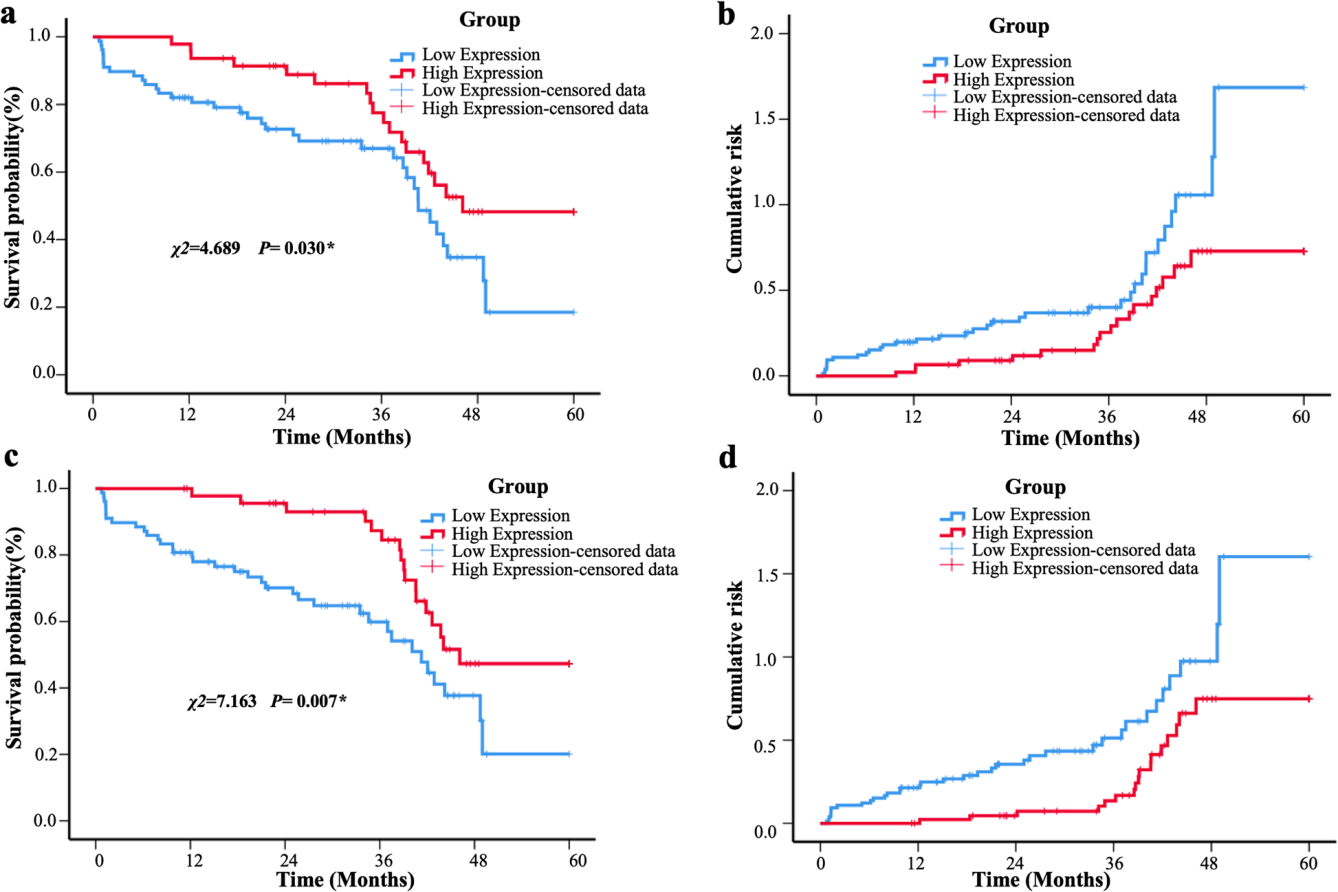
Low expression of PINK1 and PARK2 predicts poor prognosis in patients with ESCC. **a** The Kaplan–Meier (K-M) survival curve shows that patients with ESCC who have low PINK1 expression have a poor prognosis (*P* = 0.030). **b** The cumulative risk curve shows high-risk ESCC deaths associated with low PINK1 expression. **c** The K-M survival curve shows patients with ESCC who have low PARK2 expression have a poor prognosis (*P* = 0.007). **d** The cumulative risk curve shows high-risk ESCC deaths associated with low PARK2 expression

We have the clinical follow-up data on 125 patients. The Kaplan–Meier analysis was used to determine the relationship between PINK1 and PARK2 expression and OS in patients with ESCC. Patients with ESCC with low PINK1 expression had significantly lower OS than those with high expression (*P* = 0.030, Fig. 2a and b). Patients with low expression of PARK2 also had a worse OS (*P* = 0.007, Fig. 2c and d).

**Fig. 3:**
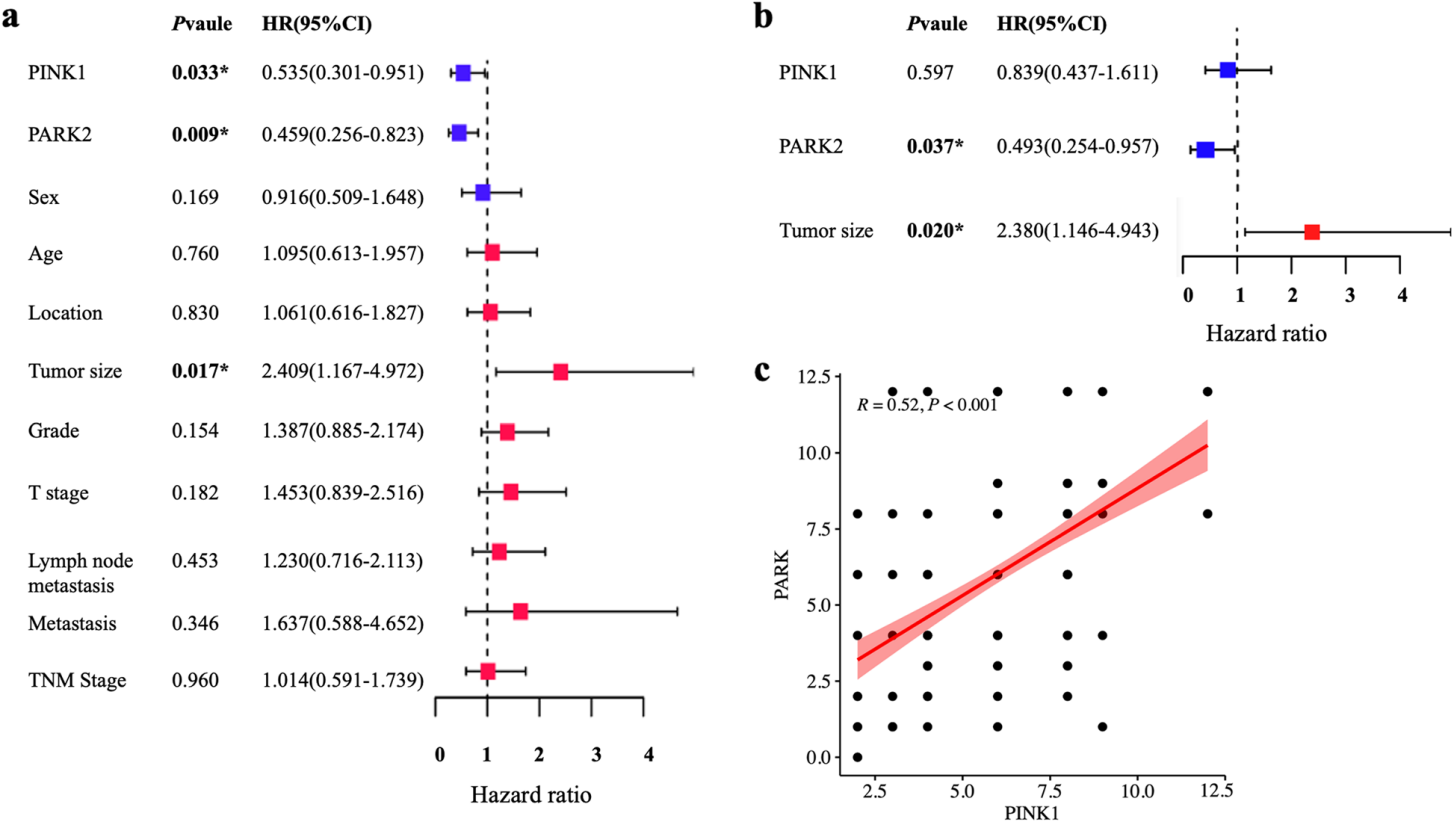
Cox univariate and multivariate analyses of prognostic factors in patients with ESCC and PINK1 and PARK2 proteins are closely related. **a** Cox univariate regression analysis. **b** Cox multivariate regression analysis. **c** Spearman’s correlation analysis showed that the PINK1 and PARK2 proteins were closely related

The clinical features were examined using Schoenfeld residuals to determine whether they adhered to the Cox regression assumptions. The results showed that the variables followed the Cox regression assumptions (Supplementary Fig. 1). Cox univariate and multivariate analyses were performed to determine the risk factors affecting the OS of patients with ESCC, including sex, age, location, tumor size, grade, T stage, lymph node metastasis, metastasis, TNM stage, and PINK1 and PARK2 expression patterns. The result shows that patients with ESCC who had reduced PINK1, PARK2, and tumor size had worse prognoses (Fig. 3a). The low-PINK1 group patients with ESCC had significantly lower survival (95% confidence interval (CI) = 0.302–0.951, *P* = 0.033). Patients with low PARK2 expression had worse OS (95% CI = 0.256–0.823, *P* = 0.009). Tumor size was correlated with OS (95% CI = 1.167–4.972, *P* = 0.017). However, the age, sex, location, grade, T stage, lymph node metastasis, metastasis, and TNM stage were not. Furthermore, Cox multivariate analysis revealed that low PARK2 expression was correlated with poor OS and that low PARK2 expression in ESCC was independently predictive of poor OS (95% CI = 0.254–0.957, *P* = 0.037; Fig. 3b).

### PINK1 was related to PARK2 and had a similar signaling pathway to PARK2

**Fig. 4:**
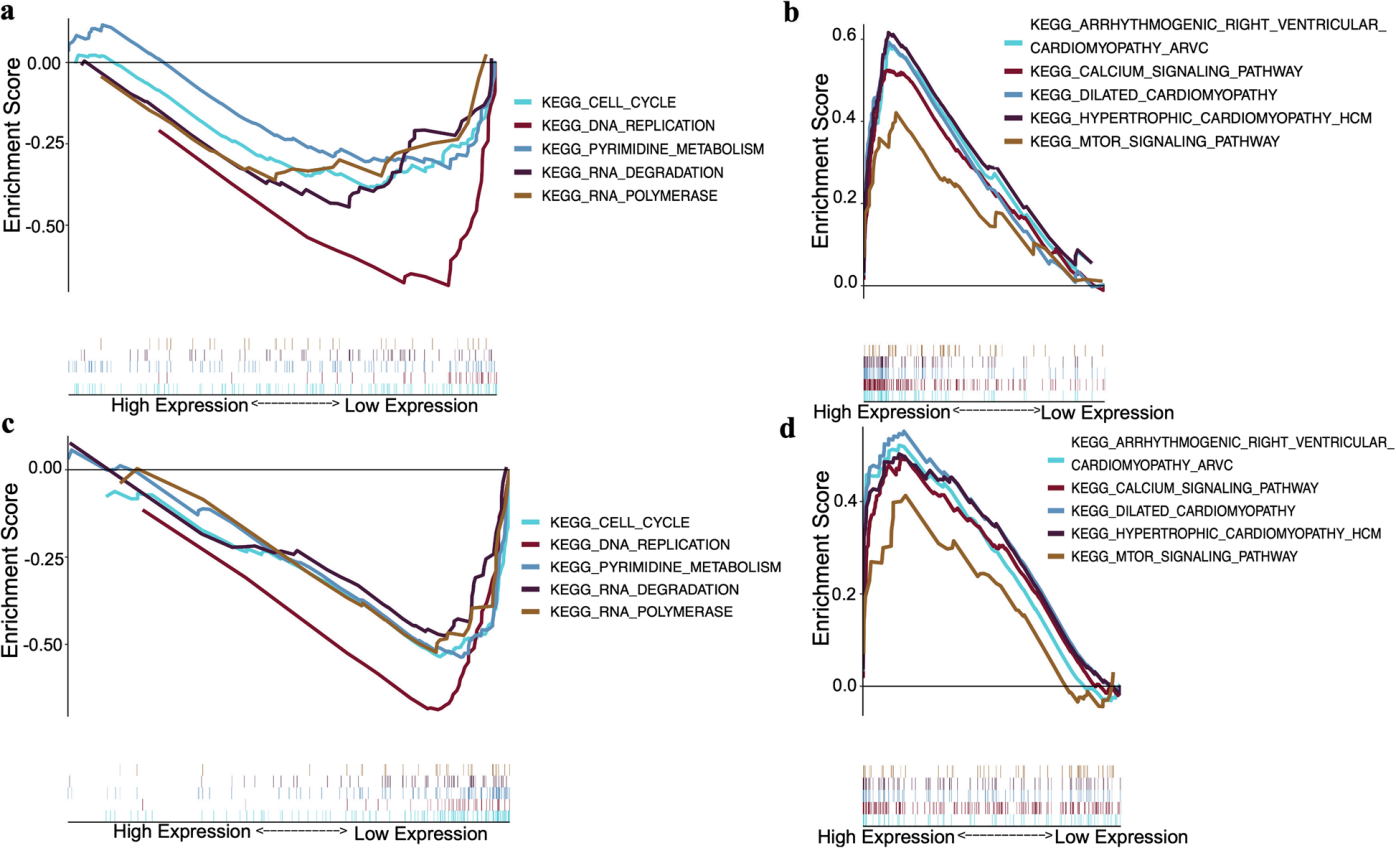
PINK1 and PARK2 share pathways. **a** KEGG pathways enriched in the PINK1 low-expression group. **b** KEGG pathways enriched in the PINK1 high-expression group. **c** KEGG pathways enriched in the PARK2 low-expression group. **d** KEGG pathways enriched in the PARK2 high-expression group

Spearman’s correlation analysis showed that the PINK1 and PARK2 proteins were related (*R* = 0.520, *P* < 0.001, Fig. 3c). Furthermore, the gene expression profiles of 178 EC samples were collected from The Cancer Genome Atlas (TCGA) database, and GSEA was used to analyze the functional enrichment in the high and low PINK1 and PARK2 expression groups (Fig. 4). KEGG pathway enrichment revealed that the low-expression of PINK1 and PARK2 groups shared the same signal pathways, which were primarily associated with cell cycle, DNA replication, and pyrimidine metabolism (Figs. 4a and c), whereas the high-expression groups were mainly associated with the calcium signal pathway and the mTOR signal pathway (Figs. 4b and d).

## Discussion

PINK1 targets the outer mitochondrial membrane [31]. It controls mitophagy, cell proliferation, apoptosis, and angiogenesis [25, 32]. PINK1 promotes tumor cell proliferation, apoptosis, migration, and invasion in breast [20], gastric [33], colorectal, and liver carcinomas [20]. Abnormal PINK1 expression promotes tumor cell proliferation and invasion indefinitely.

Our findings showed that ESCC had low PINK1 expression. ESCC had 37.5% PINK1 overexpression, compared to 48.4% in adjacent normal tissues. Oncogenesis and tumor development involve mitophagy [25, 33]. Mitophagy involves the PINK1 protein. Xu et al. [34] found that PINK1 downregulation increased gastric cancer cell proliferation and migration by suppressing mitophagy and increasing mitochondrial reactive oxygen species (ROS). Our findings indicated that PINK1 protein expression was associated with the grade, T stage, and TNM stage but not with age, sex, location, tumor size, lymph node metastasis, or metastasis. The low-expressed PINK1 group ESCC had worse differentiation. Patients with advanced T stage and advanced TNM stage had reduced PINK1 expression. Thus, PINK1 could be an ESCC biomarker.

PINK1 modulates immune reactions [35], metabolism [36], and chemosensitivity [37], making it a potential prognostic marker. According to Agnihotri et al. [36], PINK1 inhibits glioblastoma cell growth by regulating the Warburg effect. PINK1 targets the intervention of mitogen-cox-2/drp1-dependent mitochondrial dynamics and increases hepatocellular carcinoma chemosensitivity [37]. Furthermore, PINK1 can directly induce metformin and arsenic trioxide synergy in cervical cancer [38]. The survival curve in our study showed that patients with low-PINK1 ESCC had a shorter OS. In Cox univariate analysis, PINK1 was a prognostic risk factor for patients with ESCC. PINK1 was found to be elevated PINK1 was found to be elevated in non-small cell lung cancer and was linked to mitochondrial dysfunction. Furthermore, high PINK1 expression predicts a poor prognosis [20, 39]. However, due to some patients losing follow-up, these findings are limited (among 232 patients, 125 were followed up). All patients underwent radical resection of ESCC. However, some patients underwent thoracic surgery for radical resection, and others underwent thoracic laparoscopic assisted radical resection. Meanwhile, individual patients were infected with bacteria after surgery, which may affect their prognosis. It should also be confirmed that low expression of PINK1 is associated with shorter survival in a larger sample of patients.

PARK2, an E3 ubiquitin ligase, is suppressed in the cytoplasm. During environmental-induced mitochondrial autophagy, PARK2 in the cytoplasm activates and recruits into the mitochondria to improve mitochondrial quality control [22]. PARK2 protein, a transcription regulator, plays multiple roles in different diseases. PARK2 reduces serine production and glycolysis, which inhibit tumor cell proliferation [28, 30]. In contrast to normal tissues (48.4%), ESCC tissues had 60.8% PARK2 low expression. So, PARK2 was lowly expressed in ESCC. We also found that lymph node metastasis and TNM stage were linked to PARK2 protein expression. Patients with lymph node metastases and an advanced TNM stage expressed less PARK2. The survival curve in our study revealed that patients with ESCC who had low PARK2 expression had a shorter OS, and Cox univariate and multivariate analyses revealed that low PARK2 expression independently predicted the poor OS in ESCC. These findings are consistent with a previous study on non-small cell lung cancer [28].

Members of the PD gene family, PINK1 and PARK2, regulate mitochondrial autophagy, turnover, and biogenesis [40]. Their abnormalities cause familial Parkinson’s disease [10]. They regulate mitochondrial autophagy through the PINK1/Parkin signal pathway [40]. Parkin deficiency-induced PINK1 inactivation causes cancer [41]. Parkin and PINK1 deletions synergistically promote tumor development [41]. Our study showed that the low expression of PINK1 and PARK2 proteins was strongly associated with the prognosis of patients with ESCC. Moreover, the functional enrichment analysis revealed that PINK1 and PARK2 shared the same signal pathways. Because PINK1 and PARK2 were low expressed in ESCC, we focused on the KEGG pathway enrichment in the lowly expressed group. We found that the signal pathways were cell cycle, DNA replication, and pyrimidine metabolism. In Kras-driven pancreatic ductal adenocarcinoma (PDAC), the deletion of PINK1 and PARK2 increased cell proliferation and metastasis [14, 42]. PINK1 and PARK2 also destabilize HIF-1ɑ, with PARK2 promoting its degradation [43]. In PDAC, the deletion of PINK1 or PARK2 reverses HIF-1ɑ deletion, increasing glycolysis and ROS [42]. Some amino acids, such as aspartic acid, require functional mitochondria for nucleotide biosynthesis, which promotes tumor cell proliferation [44]. Parkin or PINK1 deletion impairs mitophagy and amino acid biosynthesis [28]. PARK2 expression, according to Gupta A et al. [41], increases PINK1, which inhibits the PI3K/AKT signaling pathway, reducing cell survival and proliferation. Therefore, the PINK1/PARK2 signaling pathway may regulate the glucose metabolism of tumors by regulating mitochondrial function, thereby regulating tumor proliferation and metastasis and mediating tumor prognosis. However, further research is needed to illuminate the regulatory mechanism of the PINK1/ PARK2 signaling pathway in ESCC.

## Conclusions

PINK1 and PARK2 are risk factors for ESCC patients and may be tumor suppressors. The PINK1 and PARK2 proteins were closely related and shared the same biological signaling pathways. The PINK1/PARK2 signal pathway may represent a potential target for ESCC treatment.

### Supplementary Information

**Additional file 1. **

## Data Availability

The datasets used and analyzed during the current study are available from the author upon reasonable request.

## Abbreviations

PD: Parkinson’s disease
ESCC: Esophageal squamous cell carcinoma
EC: Esophageal carcinoma
TNM: Tumor node metastasis
OS: Overall survival
IHC: Immunohistochemistry
GSEA: Gene set enrichment analysis
KEGG: Kyoto Encyclopedia of Genes and Genomes
CI: Confidence interval
PDAC: Pancreatic ductal adenocarcinoma

## References

[1] Sung H, Ferlay J, Siegel RL, Laversanne M, Soerjomataram I, Jemal A, Bray F (2021). Global Cancer Statistics 2020: GLOBOCAN estimates of incidence and mortality worldwide for 36 cancers in 185 countries. CA Cancer J Clin.

[2] Arnold M, Soerjomataram I, Ferlay J, Forman D (2015). Global incidence of oesophageal cancer by histological subtype in 2012. Gut.

[3] Zhao Y, Zhao W, Li J, Lin S, Li L, Ren Z, Lu J, Xing X, Liu X (2022). Effect of dietary consumption on the survival of esophageal squamous cell carcinoma: a prospective cohort study. Eur J Clin Nutr..

[4] Chen Y, Ye J, Zhu Z, Zhao W, Zhou J, Wu C, Tang H, Fan M, Li L, Lin Q (2019). Comparing paclitaxel plus fluorouracil versus cisplatin plus fluorouracil in chemoradiotherapy for locally advanced esophageal squamous cell cancer: a randomized, multicenter, phase III clinical trial. J Clin Oncol.

[5] Wu C, Wang Z, Song X, Feng XS, Abnet CC, He J, Hu N, Zuo XB, Tan W, Zhan Q (2014). Joint analysis of three genome-wide association studies of esophageal squamous cell carcinoma in Chinese populations. Nat Genet.

[6] Lesseur C, Ferreiro-Iglesias A, McKay JD, Bosse Y, Johansson M, Gaborieau V, Landi MT, Christiani DC, Caporaso NC, Bojesen SE (2021). Genome-wide association meta-analysis identifies pleiotropic risk loci for aerodigestive squamous cell cancers. PLoS Genet.

[7] Chang J, Tan W, Ling Z, Xi R, Shao M, Chen M, Luo Y, Zhao Y, Liu Y, Huang X (2017). Genomic analysis of oesophageal squamous-cell carcinoma identifies alcohol drinking-related mutation signature and genomic alterations. Nat Commun.

[8] Lin Y, Chen Q, Liu QX, Zhou D, Lu X, Deng XF, Yang H, Zheng H, Qiu Y (2018). High expression of DJ-1 promotes growth and invasion via the PTEN-AKT pathway and predicts a poor prognosis in colorectal cancer. Cancer Med.

[9] Panicker N, Ge P, Dawson VL, Dawson TM (2021). The cell biology of Parkinson's disease. J Cell Biol.

[10] Ejma M, Madetko N, Brzecka A, Guranski K, Alster P, Misiuk-Hojlo M, Somasundaram SG, Kirkland CE, Aliev G (2020). The links between Parkinson’s disease and cancer. Biomedicines.

[11] Wang M, Luan S, Fan X, Wang J, Huang J, Gao X, Han D (2022). The emerging, multifaceted role of PINK1 in cancer biology. Cancer Sci.

[12] Kia DA, Zhang D, Guelfi S, Manzoni C, Hubbard L, Reynolds RH, Botia J, Ryten M, Ferrari R, Lewis PA (2021). Identification of candidate Parkinson disease genes by integrating genome-wide association study, expression, and epigenetic data sets. JAMA Neurol.

[13] Hardy J (2010). Genetic analysis of pathways to Parkinson disease. Neuron.

[14] Matsuda S, Nakanishi A, Minami A, Wada Y, Kitagishi Y (2015). Functions and characteristics of PINK1 and Parkin in cancer. Front Biosci (Landmark Ed).

[15] Li C, Pan Y, Tan Y, Wang Y, Sun X (2022). PINK1-dependent mitophagy reduced endothelial hyperpermeability and cell migration capacity under simulated microgravity. Front Cell Dev Biol.

[16] Beilina A, Van Der Brug M, Ahmad R, Kesavapany S, Miller DW, Petsko GA, Cookson MR (2005). Mutations in PTEN-induced putative kinase 1 associated with recessive parkinsonism have differential effects on protein stability. Proc Natl Acad Sci U S A.

[17] Berthier A, Navarro S, Jimenez-Sainz J, Rogla I, Ripoll F, Cervera J, Pulido R (2011). PINK1 displays tissue-specific subcellular location and regulates apoptosis and cell growth in breast cancer cells. Hum Pathol.

[18] Liu Y, Lear TB, Verma M, Wang KZ, Otero PA, McKelvey AC, Dunn SR, Steer E, Bateman NW, Wu C (2020). Chemical inhibition of FBXO7 reduces inflammation and confers neuroprotection by stabilizing the mitochondrial kinase PINK1. JCI Insight.

[19] Satoh K, Yachida S, Sugimoto M, Oshima M, Nakagawa T, Akamoto S, Tabata S, Saitoh K, Kato K, Sato S (2017). Global metabolic reprogramming of colorectal cancer occurs at adenoma stage and is induced by MYC. Proc Natl Acad Sci U S A.

[20] Li J, Xu X, Huang H, Li L, Chen J, Ding Y, Ping J (2022). Pink1 promotes cell proliferation and affects glycolysis in breast cancer. Exp Biol Med (Maywood).

[21] Yamashita K, Miyata H, Makino T, Masuike Y, Furukawa H, Tanaka K, Miyazaki Y, Takahashi T, Kurokawa Y, Yamasaki M (2017). High expression of the mitophagy-related protein Pink1 is associated with a poor response to chemotherapy and a poor prognosis for patients treated with neoadjuvant chemotherapy for esophageal squamous cell carcinoma. Ann Surg Oncol.

[22] Wang XL, Feng ST, Wang ZZ, Yuan YH, Chen NH, Zhang Y (2021). Parkin, an E3 ubiquitin ligase, plays an essential role in mitochondrial quality control in Parkinson's disease. Cell Mol Neurobiol.

[23] Johnson BN, Berger AK, Cortese GP, Lavoie MJ (2012). The ubiquitin E3 ligase parkin regulates the proapoptotic function of Bax. Proc Natl Acad Sci U S A.

[24] Lee MH, Cho Y, Jung BC, Kim SH, Kang YW, Pan CH, Rhee KJ, Kim YS (2015). Parkin induces G2/M cell cycle arrest in TNF-alpha-treated HeLa cells. Biochem Biophys Res Commun.

[25] De Snoo ML, Friesen EL, Zhang YT, Earnshaw R, Dorval G, Kapadia M, O'Hara DM, Agapova V, Chau H, Pellerito O (2019). Bcl-2-associated athanogene 5 (BAG5) regulates Parkin-dependent mitophagy and cell death. Cell Death Dis.

[26] Fujiwara M, Marusawa H, Wang HQ, Iwai A, Ikeuchi K, Imai Y, Kataoka A, Nukina N, Takahashi R, Chiba T (2008). Parkin as a tumor suppressor gene for hepatocellular carcinoma. Oncogene.

[27] Veeriah S, Taylor BS, Meng S, Fang F, Yilmaz E, Vivanco I, Janakiraman M, Schultz N, Hanrahan AJ, Pao W (2010). Somatic mutations of the Parkinson's disease-associated gene PARK2 in glioblastoma and other human malignancies. Nat Genet.

[28] Duan H, Lei Z, Xu F, Pan T, Lu D, Ding P, Zhu C, Pan C, Zhang S (2019). PARK2 suppresses proliferation and tumorigenicity in non-small cell lung cancer. FRONT ONCOL.

[29] Bhat ZI, Kumar B, Bansal S, Naseem A, Tiwari RR, Wahabi K, Sharma GD, Alam RM (2019). Association of PARK2 promoter polymorphisms and methylation with colorectal cancer in North Indian population. Gene.

[30] Liu J, Zhang C, Wu H, Sun XX, Li Y, Huang S, Yue X, Lu SE, Shen Z, Su X (2020). Parkin ubiquitinates phosphoglycerate dehydrogenase to suppress serine synthesis and tumor progression. J Clin Invest.

[31] Yan C, Gong L, Chen L, Xu M, Abou-Hamdan H, Tang M, Desaubry L, Song Z (2020). PHB2 (prohibitin 2) promotes PINK1-PRKN/Parkin-dependent mitophagy by the PARL-PGAM5-PINK1 axis. Autophagy.

[32] Liu K, Lee J, Kim JY, Wang L, Tian Y, Chan ST, Cho C, Machida K, Chen D, Ou JJ (2017). Mitophagy controls the activities of tumor suppressor p53 to regulate hepatic cancer stem cells. Mol Cell.

[33] Porporato PE, Filigheddu N, Pedro J, Kroemer G, Galluzzi L (2018). Mitochondrial metabolism and cancer. Cell Res.

[34] Xu Y, Lu J, Tang Y, Xie W, Zhang H, Wang B, Zhang S, Hou W, Zou C, Jiang P (2022). PINK1 deficiency in gastric cancer compromises mitophagy, promotes the Warburg effect, and facilitates M2 polarization of macrophages. Cancer Lett.

[35] Zhu L, Wu W, Jiang S, Yu S, Yan Y, Wang K, He J, Ren Y, Wang B (2020). Pan-cancer analysis of the mitophagy-related protein PINK1 as a biomarker for the immunological and prognostic role. Front Oncol.

[36] Agnihotri S, Golbourn B, Huang X, Remke M, Younger S, Cairns RA, Chalil A, Smith CA, Krumholtz SL, Mackenzie D (2016). PINK1 Is a negative regulator of growth and the Warburg effect in glioblastoma. Cancer Res.

[37] Che L, Wu JS, Du ZB, He YQ, Yang L, Lin JX, Lei Z, Chen XX, Guo DB, Li WG (2022). Targeting mitochondrial COX-2 enhances chemosensitivity via Drp1-dependent remodeling of mitochondrial dynamics in hepatocellular carcinoma. Cancers (Basel).

[38] Chen J, Zhou C, Yi J, Sun J, Xie B, Zhang Z, Wang Q, Chen G, Jin S, Hou J (2021). Metformin and arsenic trioxide synergize to trigger Parkin/pink1-dependent mitophagic cell death in human cervical cancer HeLa cells. J Cancer.

[39] Lu X, Liu QX, Zhang J, Zhou D, Yang GX, Li MY, Qiu Y, Chen Q, Zheng H, Dai JG (2020). PINK1 overexpression promotes cell migration and proliferation via regulation of autophagy and predicts a poor prognosis in lung cancer cases. Cancer Manag Res.

[40] Truban D, Hou X, Caulfield TR, Fiesel FC, Springer W (2017). PINK1, Parkin, and mitochondrial quality control: what can we learn about Parkinson's disease pathobiology?. J Parkinsons Dis.

[41] Gupta A, Anjomani-Virmouni S, Koundouros N, Dimitriadi M, Choo-Wing R, Valle A, Zheng Y, Chiu YH, Agnihotri S, Zadeh G (2017). PARK2 depletion connects energy and oxidative stress to PI3K/Akt activation via PTEN S-nitrosylation. Mol Cell.

[42] Li C, Zhang Y, Cheng X, Yuan H, Zhu S, Liu J, Wen Q, Xie Y, Liu J, Kroemer G (2018). PINK1 and PARK2 suppress pancreatic tumorigenesis through control of mitochondrial iron-mediated immunometabolism. Dev Cell.

[43] Liu J, Zhang C, Zhao Y, Yue X, Wu H, Huang S, Chen J, Tomsky K, Xie H, Khella CA (2017). Parkin targets HIF-1alpha for ubiquitination and degradation to inhibit breast tumor progression. Nat Commun.

[44] Elliott IA, Dann AM, Xu S, Kim SS, Abt ER, Kim W, Poddar S, Moore A, Zhou L, Williams JL (2019). Lysosome inhibition sensitizes pancreatic cancer to replication stress by aspartate depletion. Proc Natl Acad Sci U S A.

