# Supplementary figures and images for: Low expression of PINK1 and PARK2 predicts poor prognosis in patients with esophageal squamous cell carcinoma
Source: World J Surg Oncol. 2023 Oct 13;21:321. doi: 10.1186/s12957-023-03206-3 (PMC10571472; doi:10.1186/s12957-023-03206-3)

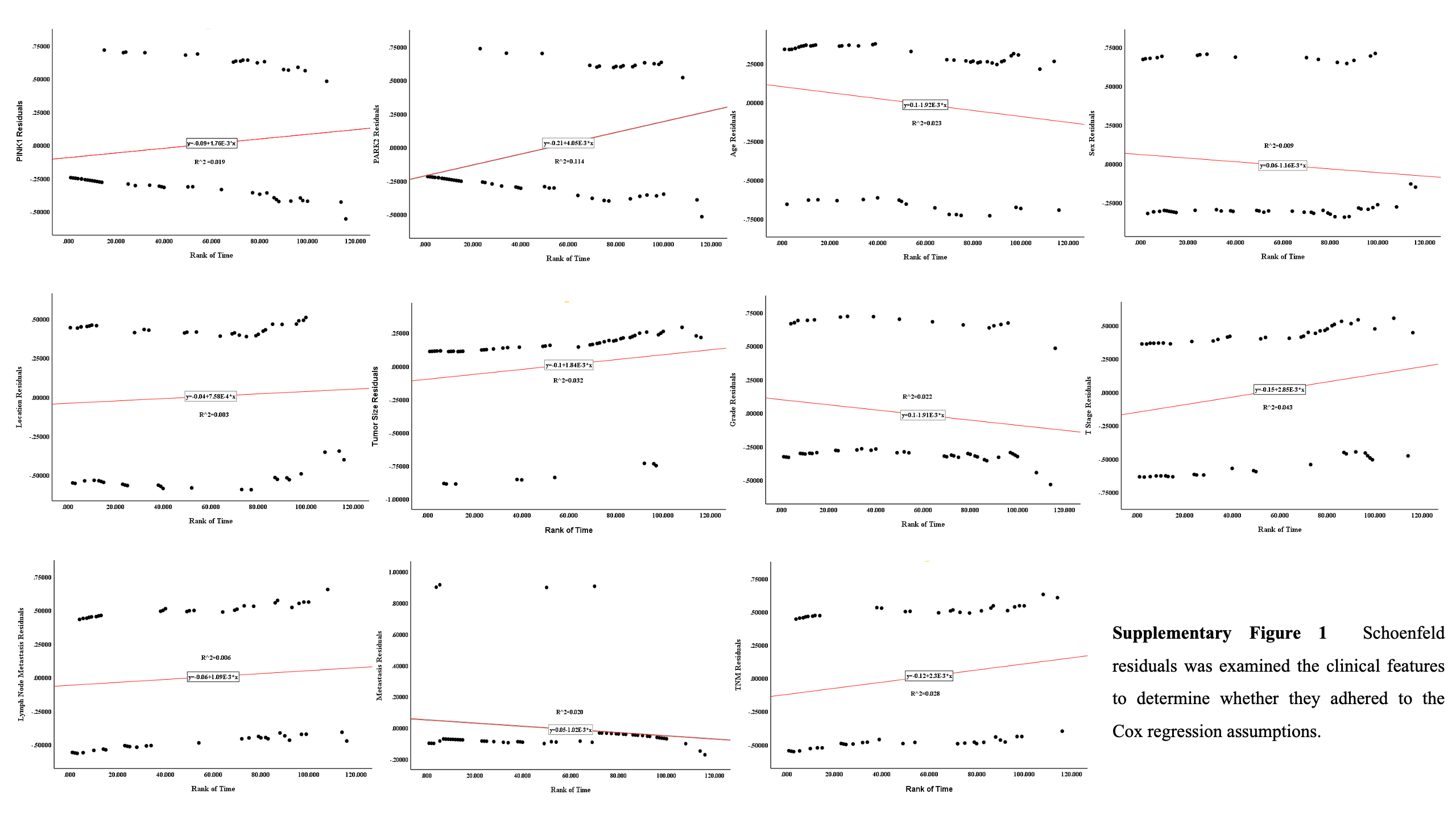

Supplement: Supplementary file 1 — Additional file 1. [file 12957_2023_3206_MOESM1_ESM.png]
